# Freeze-Drying of Plant Tissue Containing HBV Surface Antigen for the Oral Vaccine against Hepatitis B

**DOI:** 10.1155/2014/485689

**Published:** 2014-10-12

**Authors:** Marcin Czyż, Radosław Dembczyński, Roman Marecik, Justyna Wojas-Turek, Magdalena Milczarek, Elżbieta Pajtasz-Piasecka, Joanna Wietrzyk, Tomasz Pniewski

**Affiliations:** ^1^Institute of Plant Genetics, Polish Academy of Sciences, Strzeszyńska 34, 60-479 Poznań, Poland; ^2^Poznań University of Life Sciences, Wojska Polskiego 28, 60-995 Poznań, Poland; ^3^Institute of Immunology and Experimental Therapy, Polish Academy of Sciences, Rudolfa Weigla 12, 53-114 Wrocław, Poland

## Abstract

The aim of this study was to develop a freeze-drying protocol facilitating successful processing of plant material containing the small surface antigen of hepatitis B virus (S-HBsAg) while preserving its VLP structure and immunogenicity. Freeze-drying of the antigen in lettuce leaf tissue, without any isolation or purification step, was investigated. Each process step was consecutively evaluated and the best parameters were applied. Several drying profiles and excipients were tested. The profile of 20°C for 20 h for primary and 22°C for 2 h for secondary drying as well as sucrose expressed efficient stabilisation of S-HBsAg during freeze-drying. Freezing rate and postprocess residual moisture were also analysed as important factors affecting S-HBsAg preservation. The process was reproducible and provided a product with VLP content up to 200 *µ*g/g DW. Assays for VLPs and total antigen together with animal immunisation trials confirmed preservation of antigenicity and immunogenicity of S-HBsAg in freeze-dried powder. Long-term stability tests revealed that the stored freeze-dried product was stable at 4°C for one year, but degraded at elevated temperatures. As a result, a basis for an efficient freeze-drying process has been established and a suitable semiproduct for oral plant-derived vaccine against HBV was obtained.

## 1. Introduction

Effective subunit vaccines against hepatitis B virus (HBV), based on the virus surface antigen (HBsAg), were introduced over 25 years ago enabling implementation of hepatitis B mass vaccination programmes. Still, the number of chronic carriers and postdisease mortality around the world, particularly in developing countries, have been steadily growing [1, 2]. The HBV epidemiological situation has been and still does provide a rationale for ongoing research on easy to administer, plant-based vaccines against HBV.

Research focused on the development of an oral plant-based vaccine against HBV has been conducted for over 15 years. Although initial experiments provided positive results as orally delivered raw plant tissue bearing HBsAg induced a specific humoral response [3], it was outlined that the future oral vaccine has to be formulated from processed plant material, most adequately via lyophilisation [4, 5]. Freeze-dried formulations facilitate elimination of complex material preparation, size reduction, and better stability during storage, as well as easy handling and controlled administration regime. This is highly attractive regarding priorities of efficacious, cost-effective, and reliable mass hepatitis B vaccination programmes in developing countries [1, 2, 6, 7]. Preliminary trials proved that freeze-dried material containing the small surface antigen of HBV (S-HBsAg) without exogenous adjuvants induced a systemic immune response above the nominal protective titre in mice. Nevertheless, lyophilisation of plant material required further investigation since 90% degradation of S-HBsAg in the exactly immunogenic form, assembled into virus-like particles (VLPs), was observed during that process [4].

Freeze-drying, although being a well-established method commonly used for pharmaceuticals, is a process still subjected to empirical practice [8, 9]. This method having a great potential for improvement stability of labile substances, especially proteins [10] and liposomes [11, 12], also generates physiochemical stresses, which can denature proteins to various degree. Even after successful lyophilisation, the obtained product may still have limited long-term storage stability. Both lyophilisation parameters and material formulation must therefore be determined to obtain a high quality product. In general, a freeze-drying process comprises three stages: freezing (material solidification), primary drying (ice sublimation), and secondary drying (bound water desorption) [9]. To preserve protein from denaturation caused by freezing (cryoprotection) and/or dehydration (lyoprotection), a stabilising excipient(s) may be used, in parallel to the established optimal process profile [8–10, 13], among which sugars and polyols are the most common [10, 13–16]. These can serve both as the amorphous phase serving protection for an active agent or as a bulking component providing desired physical properties of a lyophilised solid, sometimes expressing those abilities in parallel.

There are two main hypotheses explaining protein stabilisation during drying and storage. One is the “glass dynamics hypothesis” [17] which states that a stabiliser forms a rigid, inert matrix into which the protein is molecularly immobilised during freezing. Therefore, stabilisation is via a purely kinetic mechanism; limited mobility prevents unfolding and other degradations. Another is the “water substitute hypothesis” [18–20] in which a stabiliser can form hydrogen bonds at specific sites on protein surface and substitute for the thermodynamic stabilisation function of water that is lost during drying. Therefore, unfolding of the protein is inhibited thermodynamically during the drying process by an amorphous component. Such binding takes place somewhere during the secondary drying when bound water is being removed [21].

The aim of this study was to develop a stable dry-powder formulation containing S-HBsAg VLPs expressed in previously obtained lettuce [4], with the objective to preserve its structural integrity, antigenicity and immunogenicity. The freeze-drying parameters together with added excipients were investigated in order to select the optimal process profile to obtain a potent formula of a semiproduct for a plant-derived oral vaccine against hepatitis B.

## 2. Materials and Methods

### 2.1. Plant Material

Plant material used in the studies came from previously obtained lettuce expressing S-HBsAg [4]. Only leaves from plants of the antigen content at least 10 *μ*g/g FW were harvested for lyophilisation experiments.

### 2.2. Freeze and Thaw Tests

Two freezing methods, “fast” and “slow”, were tested on leaf blades. “Fast” freezing was performed by submerging the material into liquid nitrogen and immediately placing on a freeze-dryer shelf precooled to −35°C. The material subjected to “slow” freezing was put into a freeze-dryer chamber at ambient temperature and frozen at 2°C/min rate until −35°C. After an hour frozen samples were divided and subjected to different thawing protocols, “slow” in air of 4°C, or “fast” by immersing in 4°C water. The experiment was conducted in three replications.

### 2.3. Preparation, Lyophilisation, and Processing of Plant Material

Leaf blades for lyophilisation were infiltrated with an excipient by placing the material in a vacuum chamber and lowering pressure to 50–200 mbar maintained for 2–15 min. Then a solution of a given excipient (see Section 3) was introduced and remained for soaking to complete. The material was then placed in a layer of max. 5 mm thickness in aluminium trays on freeze-drier shelves, frozen, and freeze-dried (BETA 1–16, CHRIST, Germany) in vacuum at 0.2 mbar and under different temperatures and times for primary and secondary drying (see Section 3). Lyophilised tissue was then powdered in a coffee mill chilled to 4°C and stored in the dark at 4, 22, or 37°C in tightly sealed bottles. Residual moisture (RM) in preparations was determined using the gravimetric method [22].

Since the absolute content of total or VLP-assembled S-HBsAg in the freeze-dried tissue depended on its original level in the plants, lyophilisation effectiveness was expressed as relative antigen preservation calculated as a ratio of the antigen content in processed tissue to the fresh one multiplied by the degree of weight loss. Each experimental variant was tested in at least three replications and analysed statistically (the variance for one-way classification with the Duncan test, *P* ≤ 0.05) using Statistica 8 software (StatSoft). Since many variables were investigated, the studies were conducted in a mode, in which only the most efficient variant of a given parameter tested in the current round of experiments was used for the next round.

### 2.4. ELISA Assays of S-HBsAg

The content of S-HBsAg, both total and VLP-formed, was assayed in lettuce leaves and in derived lyophilised tissue using quantitative sandwich ELISA tests performed according to typical guidelines. Samples were ground with extraction buffer, added at a ratio of 25 : 1 for leaves and 62.5 : 1 for lyophilised tissue and consisting of PBS at pH 7.4, 10 mM Na_2_SO_3_, 2% (w/v) PVP_40000_, and 0.2% (w/v) BSA and supplemented with 0.05% (v/v) Triton X-100. Crude extracts were centrifuged for 10 min at 10.000 rpm at 4°C. The S-HBsAg content, both total and assembled into VLPs, was assayed using a MaxiSorp microplate (NUNC) coated with 0.5 *μ*g/mL anti-SHBsAg IgG2a monoclonal antibody (mAb) (Cat. Number C01455 M, Meridian Life Science) in carbonate buffer at pH 9.6. After washing with PBS containing 0.05% (v/v) Tween 20, plates were blocked with 5% (w/v) PBS-fat-free milk at 25°C for 1 h. Following washing samples, extracts were added to PBS-filled wells at 1 : 1 ratio and serially diluted. After incubation at 25°C for 45 min and washing, wells were incubated with primary and secondary antibodies, specific for a given test. For the VLPs assay, 0.5 *μ*g/mL mAb IgG2b anti-SHBsAg (Cat. Number C92100 M, Meridian Life Science) and polyclonal goat anti-mouse IgG2b (Sigma), and finally 1 : 10,000 diluted rabbit anti-goat AP-conjugated antibody (Sigma) were used, all with washing between steps. Total antigen content was assayed after samples incubation with rabbit anti-SHBsAg serum (obtained after triple vaccination of a rabbit New Zealand Big breed with Engerix B vaccine, GlaxoSmithKline), followed by biotin-conjugated anti-rabbit goat polyclonal antibody (Sigma), and followed by AP-conjugated Extravidine (Sigma). The reaction with pNPP substrate (Sigma) was developed at 25°C for 1 h and absorbance at 405 nm was measured using the microplate reader, Model 680 (Bio-Rad). The S-HBsAg concentration was calculated in micrograms per gram of fresh weight (FW) or lyophilised dry weight (DW).

### 2.5. Western Blot of S-HBsAg

Leaf fragment or lyophilised tissue, approx. 50–60 mg in weight, were ground in 300 *μ*L of PBS with 0.5% (v/v) Tween 20, then Laemmli buffer with 100 mM DTT was added and samples were incubated at 65°C for 15 min. Samples were run on 12% polyacrylamide gel with 0.1% (w/v) SDS and then blotted onto a nitrocellulose membrane (Roche). The blot was blocked with 3% (w/v) BSA in TBS and then incubated with the TBS-diluted rabbit polyclonal anti-SHBsAg antibody (Cat. No. B65811R, Meridian Life Science). The AP-conjugated goat polyclonal anti-rabbit whole-molecule antibody (Sigma) followed. The molecular weight of protein bands, visualized after a reaction with the BCIP/NBT substrate (Sigma), were estimated using a protein size marker (MBI Fermentas).

### 2.6. Mouse Immunisation

Separate groups of 10–12-week-old BALB/c mice (5 per group) were primed by an intramuscular (*i.m.*) injection of Engerix B in a dose 500 ng of S-HBsAg or with PBS for control groups. Boosting was performed on the 42nd day after priming by oral administration (*p.o.)* of PBS-suspended powdered lyophilised tissue containing 50 ng of S-HBsAg VLPs, the control lyophilised tissue, or* i.m.* delivered PBS. Blood was collected 3 days before (preimmune) and 63 days after prime immunisation. Sera were collected from blood by centrifugation at 3 000 rpm at 4°C for 15 min. Anti-HBs antibodies (mIU/mL) were assayed twice using the Monolisa anti-HBs PLUS kit (Bio-Rad) and analysed statistically (the variance for one-way classification with the Duncan test, *P* ≤ 0.05) using Statistica 8 software (StatSoft).

## 3. Results and Discussion

### 3.1. Freeze-Thawing Stability of a Plant-Associated S-HBsAg

Lyophilisation first requires solidification of the material by freezing. As this often proves detrimental to protein drugs [23] and liposomes [24], therefore the effect of cooling rate on S-HBsAg inside plant tissue was investigated. Both rapid and slow freezing rates were reported to decrease recovery of proteins with specific stresses of cryoconcentration, phase separation, and cold denaturation characteristic for slow freezing, and the formation of large ice-aqueous interface occurring during rapid cooling [25]. As a result, freezing has been found to affect primary [26, 27] and secondary [28] drying rates together with significant influence on extent of product crystallinity [29] and surface area [30] as well as protein aggregation and stability [31, 32]. Common approach for protein stabilisation and shortening of the drying cycle is to decrease supercooling effect via minimising difference between melting and nucleation temperatures [33]. In plant tissue ice nucleation is observed at temperatures ranging from subzero to −12°C, depending on tissue and species [34, 35]. The initiation of ice nucleation in plant tissues is still under discussion [36], but some modifications of this effect are possible. However, these are still not routine and require further development. Extrinsic nucleation cannot be credible as ice propagation may not cross cell wall boundary. Addition of bacteria or chemicals may promote intrinsic earlier nucleation but most do not fall within current GMP guidelines. Physical operations as electrofreezing or mechanical methods are difficult to standardise and to integrate into the equipment [37]. For the above reasons, current methods of nucleation modification were not applicable in our project on low-cost oral vaccine. Instead, we focused on determination of S-HBsAg stability during freezing as an essential factor for final efficiency of vaccine preparation.

In general, two freezing protocols are employed; “fast” using liquid nitrogen, and “slow” by ramped cooling. Both methods give different effects, with normally the lowest supercooling but higher risk of protein damage due to phase separation for the “slow” cooling method [38]. We tested both methods on raw plant material; the “fast” by quenching the tissue in liquid nitrogen with process completion within max. 5 s and the “slow” by freezing at 2°C/min rate and obtained results are summarised in Table 1. For all tested variants, a significant drop in VLP structures recovery together with some build-up of total antigen content was found, but only a slight influence of thawing rate was observed. Changes of VLPs and total antigen contents were not visibly consistent, but some trend could be observed. “Slow” freezing method promoted greater VLPs preservation of 36–40% depending on the thawing rate. This coincided with a slight increase of the total S-HBsAg content, 12% and 21%, respectively, as opposed to the higher deterioration degree in response to “fast” freezing. Both higher VLPs preservation and lower total antigen build-up indicated the “slow” freezing method as appropriate for further tests.

An observed increase of the total antigen content most probably may be attributed to degradation processes, of both VLPs and the S-HBsAg polypeptide itself. Supposedly, in plants the total content of S-HBsAg comprises both VLPs and a “non-VLP” pool of antigen structures including free antigen dimers, small aggregates, and defect particles [4]. Upon stress, VLPs are believed to disintegrate and release fragments enriching the “non-VLP” pool. Given that a single VLP structure can split into multiple aggregates and dimers, the potential for increasing the total antigen concentration is large. In parallel, depending on the nature and intensity of stresses, the polypeptide chain itself, both free and on the surface of VLPs, may denature to a various extent enhancing fluctuations of the total antigen level. Knowing that VLP-assembled S-HBsAg is a crucial vaccination agent [39], an excessive change in the total antigen level is undesirable. Thus, optimisation of plant material processing had to focus on full preservation of VLPs along with minimising changes of the total antigen level.

### 3.2. The Effect of Lyoprotectants on S-HBsAg Preservation

The next step of the study was to assess excipient potency for stabilisation of S-HBsAg during the freeze-drying process. Potential substances had been considered on the basis of their reported lyoprotective properties [7, 9–11, 13, 40–42], but with regard to oral vaccine formulation requirements, such as harmless ingestion and reasonable cost [43]. Therefore, sucrose, glucose, mannitol, glycine, and glycerol at concentrations of 100, 250, and 500 mM, were chosen. For the preliminary experiments, fresh lettuce leaves were infiltrated with all excipients under 100 mbar vacuum for 5 min and lyophilised at 5°C or 20°C for primary drying and 22°C for secondary drying.

**Figure 1:**
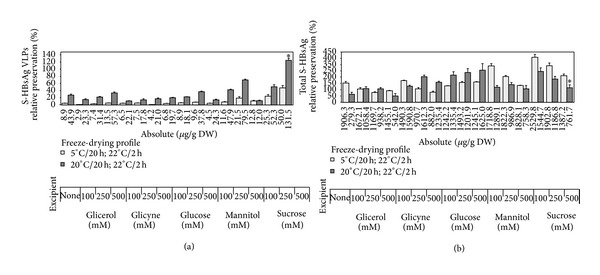
Efficiency of freeze-drying represented as preservation of S-HBsAg VLPs (a) and total (b) in lettuce leaves soaked with a series of excipients and processed under two temperature profiles. Preservation effectiveness [%] calculated as the ratio of VLP-formed and total S-HBsAg in powdered lyophilised product to the antigen content in fresh tissue multiplied by weight loss degree. Star indicates the variant selected for further experiments (see text for details).

**Figure 2:**
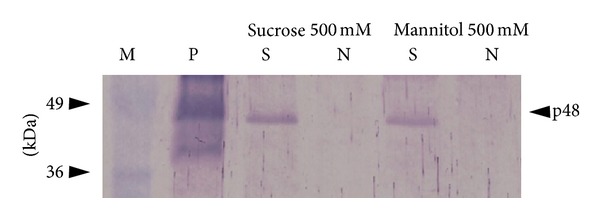
Western blot analysis of S-HBsAg in freeze-dried tissue using the specific polyclonal antibody. Lanes: M—protein molecular weight marker (MBI Fermentas), P—S-HBsAg positive control (Meridian Life Sciences). “S” and “N” analysed freeze-dried transgenic lettuce containing S-HBsAg and nontransgenic plant (negative control), respectively, soaked with a given excipient, p48—S-HBsAg dimer.

The results of the experiments (Figure 1) proved that across all tested variants, the profile with the lower shelf temperature was much less effective regarding preservation of S-HBsAg VLPs. Among excipients, sucrose and then mannitol appeared as the most suitable for lyoprotection of plant-associated S-HBsAg, as confirmed in ELISA assays and western blot analysis (Figures 1 and 2). In particular, 500 mM sucrose excelled in protecting the particled form of the antigen, both regarding absolute and relative content of VLPs and was significantly superior to the other variants (see Supplementary Table 1 in Supplementary Material available online at http://dx.doi.org/10.1155/2014/485689). After the profile with a 20°C shelf temperature, this protectant made it possible to obtain several times higher lyophilisation efficiency, in comparison to unsoaked tissue (124%). Increased VLPs level beyond 100% could be caused by partial disintegration of actual VLPs into smaller particles. Moreover, the total antigen level remained practically unchanged during that freeze-drying profile, providing a conclusion that VLPs degradation occurred only to a limited extent, oppositely to the cycle with primary drying at 5°C, where in connection with VLPs degradation the total antigen level increased to around 200%. A relatively good VLPs preservation (70%) was also observed for leaves soaked with 500 mM mannitol. However, at the same time the level of the total antigen remained unchanged, indicating that some degradation of S-HBsAg polypeptides released or aggregates released from VLPs might occur. In the other variants, low VLPs preservation together with high fluctuations of the total S-HBsAg level was observed, in most cases above the theoretical 100%. In addition to effectiveness values, some tendency was observed for VLPs preservation to increase in correlation to the increasing concentrations of excipients. This phenomenon partially coincided, especially for the variants with sucrose and mannitol, with less increased contents of total antigen. Regarding the abovementioned assumption that the higher the total antigen level the larger the pool of defect S-HBsAg forms, that effect is advantageous. Summarising this step of research, sucrose provided the best preservation of S-HBsAg in the form of VLPs and the simultaneous desirable, unchanged level of the total antigen; hence it was selected for next tests.

In addition to the improved relative effectiveness of lyophilisation, absolute VLPs contents in processed tissues were substantially higher, up to 131.5 *μ*g/g DW in the variant with 500 mM sucrose, than 11-12 *μ*g/g DW obtained previously [4]. This can facilitate a considerable increase in antigen concentration and at the same time lowering the amount of tissue needed for the preparation of a single vaccine dose, which would allow the reducuction of the amount of ballast material to be delivered and to shorten exposure of an oral vaccine to the GALT system [3, 43]. In consequence, the improved lyophilisation process can result in reducing the risk of induced oral tolerance, while at the same time making vaccine production more economically viable.

### 3.3. Freeze-Drying Associated Processing

**Figure 3:**
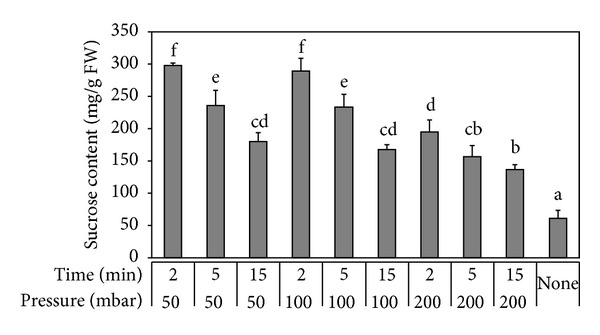
Sucrose concentration after different soaking conditions in lettuce leaf tissue. Letter indexes mark statistically homogenous groups.

Although freeze-drying was the crucial stage, associated handling steps as tissue infiltration with a lyoprotectant and milling of the product could also affect the final efficiency of whole process. Literature data as well as our results showed that the preservation effect was associated with a high concentration of a protective excipient, here 500 mM sucrose (Figure 1). Therefore maximal effectiveness of lyoprotectant infiltration had to be ensured and time (2–15 min) and pressure (50–200 mbar) of soaking were tested (Figure 3). The content of S-HBsAg was assayed after soaking, and did not show any instability in tested parameters (data not shown). What is interesting, the shortest time period proved to be the most effective in all the variants, while pressure tests revealed that under 50 mbar sucrose concentration in tissue was the highest. However, at soaking under 100 mbar it was only minimally lower and statistically equal to 50 mbar but less energy-consuming to perform. Consequently, 2 min and 100 mbar were selected as final parameters.

After freeze-drying, lyophilised tissue was milled to obtain the final semiproduct. Tests proved that within a period of 5–90 seconds, no significant differences occurred regarding both VLPs and total S-HBsAg content (data not shown). Yet, since approximately 20 seconds were enough to mill the tissue completely, this time was adopted.

### 3.4. Impact of Freeze-Drying Conditions on S-HBsAg Preservation

The successive step was focused on determining shelf temperature and duration of freeze-drying, which are of great importance for process economy and scaling up. Optimisation of freeze-drying is still a trial-based process. A higher drying temperature allows shortening cycle duration and consequently provides a more cost-effective process. Usually, each 1°C increase in product temperature decreases time of primary drying by about 13% [9, 44]. In addition, the approach of lowering the shelf temperature with prolonged drying times would result in unfeasible process regarding economical aspect, even though efficiency would be high. Therefore, an optimised freeze-drying process runs with the product temperature as high as possible [45]. However, the material cannot be overheated as collapse may occur [9, 10]. Also after-process residual moisture is an important factor, having a significant influence on protein recovery [46, 47]. In general, final parameters of the process and product formulation depend on nature of an initial material and an active protein ingredient. In our studies, nature of the material—leaf blades—and its placing in thin layer (max. 5 mm) precluded accurate readout of product temperature, thus we directly adjusted shelf temperature.

**Figure 4:**
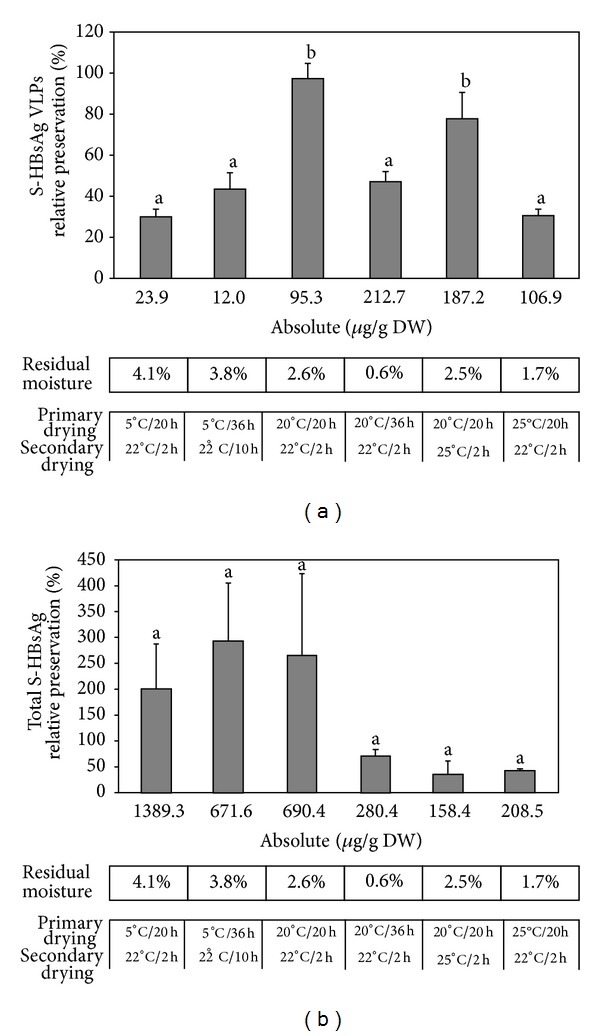
Preservation of VLP-formed (a) and total (b) S-HBsAg in plant tissue lyophilised under different temperature profiles. Sucrose at 500 mM was used as excipient in all variants. Letter indexes mark statistically homogenous groups, separately for the total and VLP-assembled S-HBsAg.

Starting point for our studies was temperature 5°C or 20°C for primary drying. The latter variant resulted in better S-HBsAg preservation, probably due to lower residual moisture of the product. Then, we decided to test higher drying temperatures to find upper limit, that is, collapse temperature, but also to prolong the process duration since it could not be excluded that low RM and S-HBsAg preservation could be achieved in that way. Tests (Figure 4) revealed that shelf temperature of 5°C resulted in much less S-HBsAg VLPs recovery, only 30%, than more intense drying at 20°C where on average 97% VLPs were recovered throughout all batches. Most probably the relatively high residual moisture of 4.1% after the profile with 5°C shelf temperature in comparison to 2.6% after the profile with 20°C was responsible for this effect. To test this, a prolonged drying time was used for the lower temperature profile. As a result, RM decreased, but efficiency improved only slightly, in addition the undesirable build-up of the total S-HBsAg occurred. The RM influence was further confirmed when a prolonged primary drying of 36 h at 20°C was applied. RM considerably lowered and a significant drop of S-HBsAg content, both VLPs and total, was observed. Next, it was assessed whether shelf temperature beyond 20°C and 22°C can be applied. When 25°C for secondary drying was used, 2.5% RM was obtained but VLPs were preserved at lower level. In addition, a sharp drop—below the VLPs level—of the total S-HBsAg occurred, probably as a result of polypeptide chain damage, both free dimers and on the surface of VLPs. Increasing the primary drying temperature to 25°C also yielded poor recovery of total as well as VLP-formed S-HBsAg, which can be a result of excessive drying conditions and low RM after the process. Eventually, the initial profile proved to be the most efficient, indicating that process conditions cannot exceed drying temperatures of 20°C and 22°C and final RM of approximately 2.6%. This profile made it possible to save around 100% of VLPs with a relatively moderate increase of the total antigen content.

Apart from the temperature profile and duration, other parameters as chamber pressure and type and nature of drying container can influence process performance. Pressure should be maintained well below the ice vapour point at the product temperature to allow high sublimation rate. But it also must be kept over certain level to avoid large heterogeneity in heat transfer and product contamination. Therefore, the optimum chamber pressure is a compromise with the moderate values of 0.15–0.2 mbar giving optimal results and with little influence on process effect within this range [48, 49]. According to literature data and practice, in this study pressure was set at 0.2 mbar. The container has to allow for optimal heat transfer between shelf and the product and avoiding limitation the water vapour rate. Hence, processed material was placed on open aluminium trays which were then placed directly on the dryer shelf and its amount of material was limited to 5 mm. These ensured no significant influence of container on drying dynamics [48].

In our study we devised primary parameters for efficient preservation of S-HBsAg during freeze-drying on a laboratory scale. For a successful scale-up of this process, it is important to convert the developed small-scale cycle parameters to a full-scale production. When transferring to commercial scale equipment, it is likely that the dryer size would be 10–100 times larger. As the heat and mass transfer coefficients will change, respectively, shelf temperature will have to be adjusted accordingly in order not to exceed the maximum product temperature. In addition, an appropriate endpoint of drying should be established for a respective equipment to secure adequate final relative moisture level [50, 51]. Another key issue is to conduct stability studies which may require addition to formula excipients such as bulking and antimicrobial agents [10]. Finally, a set of trial runs will have to be performed to efficiently develop pilot scale process cycles on target equipment [51].

### 3.5. Freeze-Drying Reproducibility and Batch-to-Batch Variation

**Figure 5:**
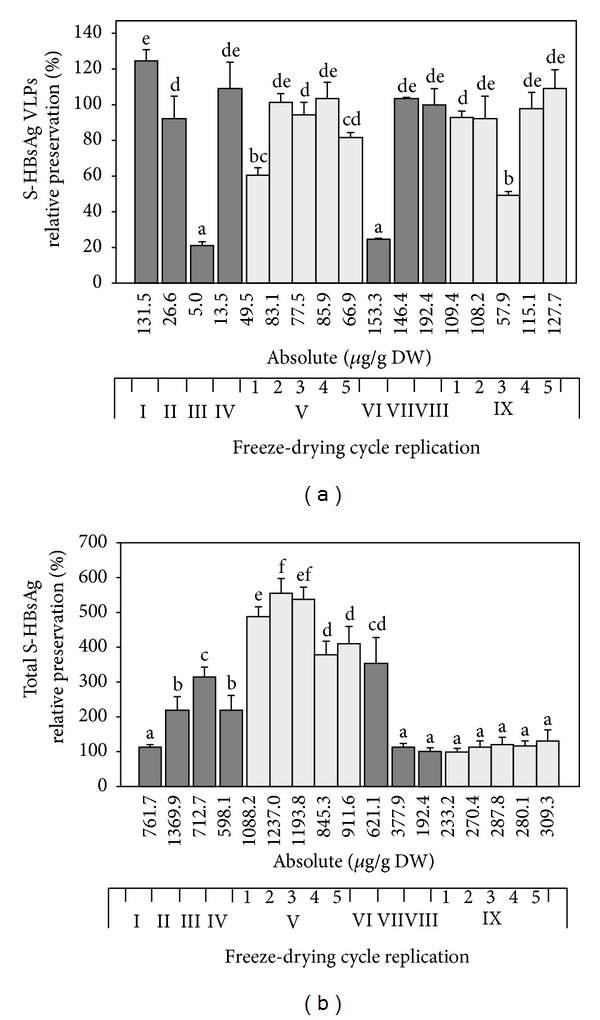
Preservation of S-HBsAg VLP-formed (a) and total (b) during freeze-drying replication tests. Each batch was run under the profile of 20°C/22 h–22°C/2 h and with 500 mM sucrose as the excipient. The Vth and IXth batches were loaded with five separate samples. Letter indexes mark statistically homogenous groups, separately for the total and VLP-assembled S-HBsAg.

The lyophilisation process is a turn-based method; hence a batch-to-batch variation does occur [9, 42]. Owing to that fact, reproducibility of the established procedure (500 mM sucrose as the excipient and the drying profile of 20°C/22 h–22°C/2 h) for plant material containing S-HBsAg was evaluated in a series of nine complete lyophilisation cycles with two cycles loaded with five separate replications (Figure 5). Preservation of VLPs remained at approximately 100%, only in three batches dropping below 50%. Batches loaded with five homogenous repetitions also exhibited fluctuations as some samples achieved 48% and 60% efficiency, proving natural process variation. Nevertheless, overall freeze-drying consistency was considered acceptable, as thirteen out of seventeen batches resulted in almost complete VLPs preservation. Even with all batches taken into account, the mean recovery of VLPs was as high as 86%. The absolute content of S-HBsAg VLPs after freeze-drying depended directly on the initial antigen level in plant material; hence the obtained values varied throughout cycles. On average powder of 100 *μ*g VLPs/g DW was achieved as plant material with ≥10 *μ*g VLPs/g FW was processed. The highest level in lyophilised material was 192.4 *μ*g/g DW when plant material with 15.9 *μ*g VLPs/g FW was used. The improved freeze-drying procedure resulted in considerable increase in the S-HBsAg VLPs content in comparison to the previous trial, where only approximately 10 *μ*g VLPs/g DW were obtained after lyophilisation of untreated material coming from plants expressing the antigen at similar level [4].

In contrast the situation regarding the total S-HBsAg level was quite different. The after-process values differed dramatically, showing no clear connection with VLPs preservation or degradation. Complete VLPs retention exhibited both an increase in the total antigen content up to 556% (batch V-2) or at an unchanged level. A possible explanation of its variation during freeze-drying might be the partial destruction of VLPs, resulting in an increase of the total antigen content, similarly as in freeze-thawing (see Section 3.1). Even though VLPs are effectively preserved during lyophilisation, some particles still can disintegrate and free S protein dimers add to the total antigen pool in the tissue. However, relatively mild process conditions posed low degradation stress on S-HBsAg polypeptide chains and resulted in maintaining the increased total antigen level. Nevertheless, the obtained absolute values of total S-HBsAg content in freeze-dried material were considerably lower than the previously reported, approximately 5000 *μ*g/g DW [4]. In this study, the total S-HBsAg level achieved a maximum 1369.9 *μ*g/g DW, but some batches resulted in values as low as 192.4 *μ*g/g DW, with the mean value for all batches at 664.2 *μ*g/g DW. Summarising, the elaborated freeze-drying process expressed satisfactory reproducibility regarding S-HBsAg preservation. Furthermore, obtained material was characterised by a high VLPs content along with the total S antigen level being not excessive, which is beneficial in terms of oral vaccination.

### 3.6. Immunogenicity Verification

**Figure 6:**
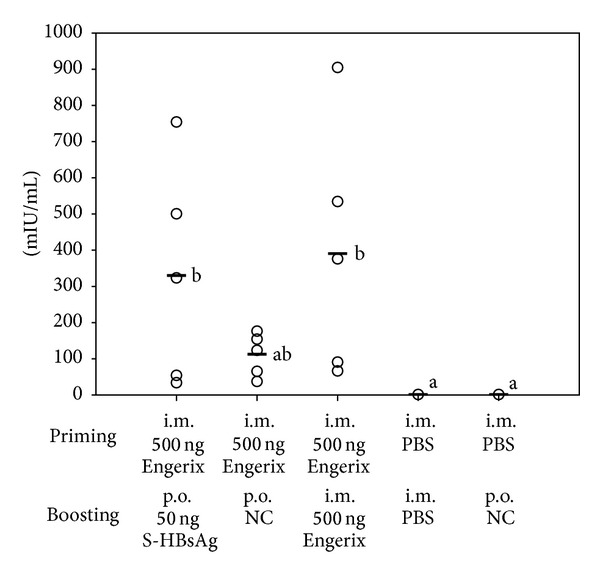
Anti-HBs antibody response in mouse serum after oral boosting with S-HBsAg in powdered lyophilised tissue. Material for immunisation was freeze-dried under the 20°C/22 h–22°C/2 h profile with 500 mM sucrose as protective excipient. Symbols: ○ individual mouse response, – group mean value. Letter indexes mark statistically homogenous groups.

To confirm the retained immunogenicity of S-HBsAg in freeze-dried plant tissue, animal immunisation trials were performed. Mice after* i.m.* priming with the Engerix B vaccine received an oral booster with powdered lyophilised tissue (see Section 2) based on the low-dose protocol reported previously [4]. Used tissue came from freeze-drying cycle number IV with 109% preservation of S-HBsAg VLPs, corresponding to 29 *μ*g/g DW and 199% or 538 *μ*g/g DW for the total antigen. Hence, the dose of 50 ng S-HBsAg was delivered in 1.72 mg of freeze-dried tissue per individual subject. Results of mouse vaccination are summarised in Figure 6. Titre of anti-HBs antibodies in mice boosted orally with lyophilised powder reached a mean value of 293 mIU/mL, while in the group boosted by injection with Engerix B they reached 397 mIU/mL. Although two of the orally boosted mice were not able to respond to preparation, the response pattern was similar to that observed for reference group boosted with Engerix B. When mice were orally delivered with the control tissue, no boosting effect was observed. Even though the used freeze-dried preparation exhibited some build-up of total S-HBsAg, apparently it did not hinder immune response development. This result might be obtained due to the considerably lower absolute level of total antigen in lyophilised tissue than that used previously [4], yet an exclusively oral immunisation pattern was then applied. The observed response, was lower but comparable to other reports on injection-oral vaccination trials. However, in those experiments the administered plant-associated S-HBsAg was CTB- or LTB-adjuvanted [52, 53]. Presented in our study induction of systemic immune response, confirmed that selected parameters of plant material processing ensure successful preservation of S-HBsAg antigenicity and immunogenicity.

### 3.7. Stability Assessment

**Figure 7:**
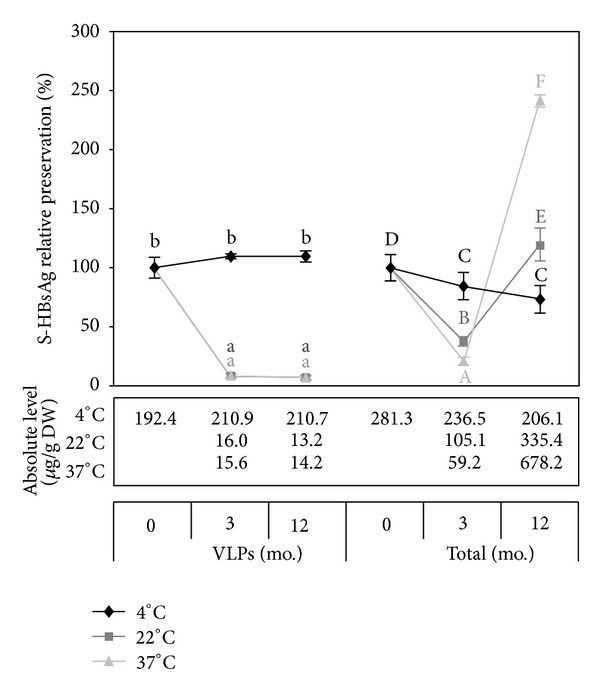
The effect of storage temperature on stability of S-HBsAg VLPs and total antigen in lyophilised tissue. Freeze-drying was performed according to the 20°C/22 h–22°C/2 h profile, with 500 mM sucrose as the protective excipient. Letter indexes mark statistically homogenous groups, separately for VLP-assembled S-HBsAg (small caps) and the total antigen (capitals).

Apart from immunogenicity preservation of plant-associated S-HBsAg, long-term stability of the antigen is crucial for vaccine design. Respective tests were conducted with sucrose soaked, freeze-dried, and powdered plant material, which was subsequently placed in sealed containers and stored for one year at three temperatures, 4°C, 22°C, and 37°C (Figure 7). The VLP-assembled antigen was stable only at the lowest temperature. The total S-HBsAg level decreased moderately during storage to a final 72% of the original value. Although this indicated that some degradation occurred, it was beneficial that no undesirable increase was observed. At higher temperatures, the VLPs content plummeted sharply (to 8%) after 3 months, but remained unchanged after that. The total antigen level also dropped substantially (below 40%) during the first 3 months, but later increased to 119% and 241% for 22°C and 37°C, respectively. This phenomenon was probably a result of delayed VLPs collapse due to its decreased rate in freeze-dried products. It is important to note, that the content of VLP-assembled S-HBsAg after the first phase of storage at 22°C or 37°C went down and subsequently remained stable at around 15 *μ*g/g DW for 8 months. These values were close to those recorded in the previous report [4] when lettuce bearing the S antigen was lyophilised without any protective excipients and the obtained preparation showed good stability over one-year storage at room temperature. It cannot be excluded that a level of 10–15 *μ*g/g of S-HBsAg VLPs in lyophilised plant material may be a natural upper limit that can be stable in the dried state at higher temperatures. Sucrose, while effective during freeze-drying itself, fails to serve as a protectant for S-HBsAg during storage at room or higher temperatures. Improvement in long-term storage at elevated temperatures, important in the aspect of vaccine distribution economy and common availability, would require further tests with a probable use of additional excipients. The obtained freeze-dried formula was stable when stored at 4°C, with the S-HBsAg VLPs content of over 200 *μ*g/g DW, several times higher than the value reported previously [4]. This provides an essential progress in terms of an oral vaccine formulation.

## 4. Conclusions

To a large extent we have improved processing via freeze-drying of plant material containing the HBV small surface antigen for the purpose of a potential oral vaccine. The procedure included excipient infiltration, freeze-drying, and powdering. The obtained formulation resulted in approx. 100% preservation of S-HBsAg assembled into immunogenic VLP structures, as well as retaining the total antigen content at the desired, not excessive, level.

Sucrose demonstrated appropriate properties as a lyoprotectant and screening of physical parameters of freeze-drying resulted in selecting appropriate shelf temperatures and drying times with regard to maintaining S-HBsAg structure and antigenicity. In addition, certain boundaries of freeze-drying parameters of shelf temperature and residual moisture were identified that cannot be exceeded to ensure the S antigen preservation. Consequently, the improved plant material processing was reproducible and made it possible to obtain the final product with a high content of S-HBsAg VLPs, up to 200 *μ*g/g DW. Finally, postprocessing preservation of native and immunogenic S-HBsAg in plant-derived preparation was confirmed during mouse immunisation trials when the orally administered preparation used as boosting elicited an immune response comparable to routine injection vaccination.

Long-term stability tests revealed that storage of the plant-derived preparation at 4°C made it possible to retain practically unchanged S-HBsAg VLPs level. However, at elevated temperatures VLPs were gradually degraded. Those results may be further expanded on studies of long-term stability at elevated temperatures, an important aspect for an easily distributed and commonly available vaccine. As a result of this research, a basis for an efficient freeze-drying process has been established and a suitable semiproduct for an oral plant derived vaccine against HBV was obtained.

## Supplementary Material

To assess excipient potency for stabilisation of S-HBsAg during the freeze-drying process series of substances were tested with regard to their reported lyoprotective properties and requirements of oral vaccine formulation. For the preliminary test, fresh lettuce leaves were infiltrated with sucrose, glucose, mannitol, glycine and glycerol at concentrations of 100, 250 and 500 mM under 100 mbar vacuum for 5 min and lyophilised at 5°C and 20°C for primary drying and 22°C for secondary drying.
Basing on absolute antigen values and relative preservation, it could be stated that across all tested variants sucrose and then mannitol at highest concentrations appeared as the most suitable for lyoprotection of S-HBsAg. Statistical analysis revealed that sucrose 500 mM was the most effective variant regarding relative content of VLPs in both drying profiles. As a consequence this protectant was chosen for further testing as expressing the best potential of S-HBsAg preservation, even when used in the unfavourable drying profile.

## Figures and Tables

**Table 1:** S-HBsAg content in transgenic lettuce leaves after freeze-thawing cycle.

Tissue		S-HBsAg			
Treatment		VLPs		Total	
Freeze	Thaw	Content [*μ*g/g FW]	Change [%]	Content [*μ*g/g FW]	Change [%]
“Fast”	“Fast”	3.4 ± 1.1	19.2 ± 6.3	39.5 ± 1.5	134.1 ± 5.2
“Fast”	“Slow”	2.9 ± 1.1	16.5 ± 5.9	40.0 ± 1.2	135.8 ± 4.0
“Slow”	“Fast”	7.0 ± 1.4	39.5 ± 8.0	33.0 ± 2.0	112.0 ± 6.8
“Slow”	“Slow”	6.3 ± 1.7	35.8 ± 9.6	35.6 ± 3.4	120.9 ± 11.7
Untreated		17.7 ± 1.6	100.0 ± 8.9	29.5 ± 1.3	100.0 ± 4.5

Material treatment: a/freezing: “fast”—liquid nitrogen quenching, “slow”—cooling at 2°C/min; b/thawing: “fast”—immersing in 4°C water, “slow”—placing in 4°C air. Percentage values represent drop of S-HBsAg compared to untreated reference sample.
