# Supplementary material for: Freeze-Drying of Plant Tissue Containing HBV Surface Antigen for the Oral Vaccine against Hepatitis B
Source: Biomed Res Int. 2014 Oct 12;2014:485689. doi: 10.1155/2014/485689 (PMC4209752; doi:10.1155/2014/485689)
Supplement: Supplementary file 1 — To assess excipient potency for stabilisation of S-HBsAg during the freeze-drying process series of substances were tested with regard to their reported lyoprotective properties and requirements of oral vaccine formulation. For the preliminary test, fresh lettuce leaves were infiltrated with sucrose, glucose, mannitol, glycine and glycerol at concentrations of 100, 250 and 500 mM under 100 mbar vacuum for 5 min and lyophilised at 5°C and 20°C for primary drying and 22°C for secondary drying. Basing on absolute antigen values and relative preservation, it could be stated that across all tested variants sucrose and then mannitol at highest concentrations appeared as the most suitable for lyoprotection of S-HBsAg. Statistical analysis revealed that sucrose 500 mM was the most effective variant regarding relative content of VLPs in both drying profiles. As a consequence this protectant was chosen for further testing as expressing the best potential of S-HBsAg preservation, even when used in the unfavourable drying profile. [file 485689.f1.docx]

**Supplementary Table**

Statistical analysis of efficiency of freeze-drying variants representing plant material treatments with a series of excipients infiltrations and processed under two drying profiles. Effectiveness represented as preservation of S-HBsAg VLPs and total antigen and calculated as the ratio [%] of VLP-formed and total S-HBsAg in powdered lyophilised product to the antigen content in fresh tissue multiplied by weight loss degree. Letter indexes mark statistically homogenous groups, separately for the VLP-assembled and total S-HBsAg.

| Profile |  |  |  |  |  | 5°C/20h | - | 22°C/2h |  |  |  |  |  | 20°C/20h | - | 22°C/2h |  |  |
| --- | --- | --- | --- | --- | --- | --- | --- | --- | --- | --- | --- | --- | --- | --- | --- | --- | --- | --- |
| S-HBsAg |  |  |  |  | **VLPs** |  |  |  | **Total** |  |  |  | **VLPs** |  |  |  | **Total** |  |
|  |  |  |  | **[%]** | **[μg/g DW]** | **statistical group** |  | **[%]** | **[μg/g DW]** | **statistical group** |  | **[%]** | **[μg/g DW]** | **statistical groups** |  | **[%]** | **[μg/g DW]** | **statistical groups** |
| Excipient | None |  |  | 4.5 ±0.8 | 8.9 ±1.7 | abcd |  | 152.2 ±12.6 | 1 906.3 ±157.4 | ghij |  | 27.9 ±3.2 | 43.9 ±4.5 | l |  | 62.1 ±17.3 | 279.3 ±77.6 | ab |
|  |  | 100 mM |  | 1.1 ±0.1 | 7.4 ±0.8 | abc |  | 104.4 ±12.6 | 1 672.1 ±202.0 | cdef |  | 15.0 ±2.3 | 23.3 ±3.6 | fgh |  | 108.4 ±14.3 | 1 058.4 ±139.8 | cdef |
|  | Glycerol | 250 mM |  | 3.1 ±0.3 | 13.5 ±2.3 | abcd |  | 77.0 ±7.4 | 1 169.7 ±112.9 | abc |  | 22.3 ±1.4 | 31.4 ±2.0 | jk |  | 106.1 ±11.4 | 938.2 ±100.5 | cdef |
|  |  | 500 mM |  | 5.3 ±0.9 | 2.9 ±0.2 | a |  | 91.5 ±3.3 | 1 455.1 ±53.2 | bcde |  | 33.1 ±3.5 | 57.3 ±6.1 | m |  | 49.9 ±20.2 | 542.0 ±219.6 | a |
|  |  | 100 mM |  | 1.3 ±0.8 | 6.5 ±1.0 | abcd |  | 171.8 ±4.0 | 1 490.3 ±34.4 | ijk |  | 11.0 ±1.1 | 22.1 ±2.1 | ef |  | 126.8 ±10.2 | 1 590.8 ±128.4 | efgh |
|  | Glycine | 250 mM |  | 4.7 ±0.1 | 7.5 ±1.2 | abcd |  | 105.8 ±11.4 | 970.7 ±104.2 | cdef |  | 14.0 ±3.1 | 17.8 ±3.9 | fg |  | 202.3 ±13.2 | 1 612.3 ±105.0 | klm |
|  |  | 500 mM |  | 5.2 ±0.7 | 4.2 ±0.3 | ab |  | 80.4 ±6.4 | 882.0 ±70.7 | abcd |  | 17.0 ±2.9 | 21.0 ±3.6 | ghi |  | 159.1 ±8.7 | 1 235.4 ±67.6 | hij |
|  |  | 100 mM |  | 4.4 ±0.2 | 6.8 ±0.3 | abcd |  | 129.1 ±3.4 | 1 242.2 ±33.1 | efgh |  | 20.0 ±1.1 | 19.7 ±1.1 | ijk |  | 215.8 ±26.3 | 1 335.4 ±162.4 | lmn |
|  | Glucose | 250 mM |  | 5.9 ±0.8 | 8.9 ±1.2 | bcd |  | 157.6 ±6.8 | 1 493.2 ±64.6 | hij |  | 22.5 ±1.1 | 18.1 ±0.9 | jk |  | 237.5 ±31.3 | 1 201.9 ±158.5 | mno |
|  |  | 500 mM |  | 7.7 ±0.5 | 9.6 ±0.6 | cde |  | 160.3 ±3.7 | 1 245.1 ±29.0 | hij |  | 37.1 ±1.6 | 37.8 ±1.6 | n |  | 254.4 ±52.6 | 1 625.0 ±335.9 | op |
|  |  | 100 mM |  | 4.8 ±0.5 | 4.5 ±0.5 | abcd |  | 289.6 ±19.0 | 1 718.8 ±113.1 | pr |  | 14.1 ±2.1 | 24.3 ±3.6 | fg |  | 119.3 ±13.7 | 1 289.1 ±148.6 | defgh |
|  | Mannitol | 250 mM |  | 8.2 ±1.6 | 11.6 ±2.2 | de |  | 204.2 ±9.3 | 1 822.3 ±82.9 | klm |  | 42.4 ±1.6 | 47.9 ±1.8 | o |  | 139.2 ±18.0 | 986.9 ±128.0 | fghi |
|  |  | 500 mM |  | 19.2 ±3.8 | 21.5 ±4.2 | hij |  | 133.8 ±1.9 | 1 828.1 ±26.4 | fghi |  | 69.8 ±2.3 | 79.5 ±2.6 | r |  | 106.1 ±28.7 | 758.3 ±205.0 | cdef |
|  |  | 100 mM |  | 11.4 ±1.6 | 12.8 ±1.8 | ef |  | 358.7 ±22.6 | 2 529.8 ±159.5 | s |  | 11.9 ±2.0 | 12.0 ±2.0 | ef |  | 245.6 ±29.5 | 1 544.7 ±185.4 | no |
|  | Sucrose | 250 mM |  | 24.3 ±4.9 | 25.3 ±5.1 | kl |  | 291.9 ±20.0 | 1 902.8 ±130.5 | r |  | 50.8 ±5.8 | 52.3 ±5.9 | p |  | 183.6 ±20.6 | 1 186.8 ±133.0 | jkl |
|  |  | 500 mM |  | 47.8 ±6.2 | 50.0 ±6.5 | p |  | 211.2 ±12.1 | 1 387.2 ±79.5 | lmn |  | 124.3 ±6.4 | 131.5 ±6.7 | s |  | 114.7 ±23.7 | 761.7 ±157.4 | cdefg |
